# Structure-Guided SOCS3 Peptidomimetics: Design and Functional Characterization

**DOI:** 10.1021/acsomega.6c01336

**Published:** 2026-04-13

**Authors:** Alessia Cugudda, Sara La Manna, Candida Bucciero, Giuliano Castellano, Anna Maria Malfitano, Daniela Marasco

**Affiliations:** † Department of Pharmacy, 9307 University of Naples Federico II, Naples 80131, Italy; ‡ Department of Translational Medical Sciences, 165474 University of Naples Federico II, Naples 80131, Italy

## Abstract

Suppressor of Cytokine Signaling 3 (SOCS3) is a pivotal negative regulator of the JAK/STAT pathway, and its loss or silencing is frequently associated with hyperactivated STAT3 signaling in aggressive cancers, including Triple-Negative Breast Cancer (TNBC). In this study, we present the rational design, biophysical characterization, and cellular evaluation of novel SOCS3-derived peptidomimetics that incorporate a hitherto unexploited structural determinant of the SOCS3/JAK2/Gp130 interface: the BC loop. The synthesis of individual and chimeric peptides was guided by structural analysis of the ternary complex, which combined the KIR/ESS regions with a stabilized BC loop. The results of the study revealed that the chimeric construct, KIRESS BC loop-chim, exhibited markedly improved affinity for JAK2 (*K*_D_ ∼ 10 μM) in comparison to the affinity of the isolated regions. This was determined by means of MicroScale Thermophoresis (MST). Circular dichroism (CD) and fluorescence spectroscopy demonstrated that turn-inducing motifs stabilize native-like conformations, correlating with enhanced serum stability. To preliminarily evaluate potential cellular effects, we assessed their serum stabilities and their cytotoxicity in MDA-MB-231 and MDA-MB-468 cells once conjugated to a small Cell-Penetreting Peptide (CPP). In both cases, the good biocompatibility of the designed mimetics appeared promising for evaluating signaling-dependent effects. These findings validate a multiregion, structure-guided design strategy and identify an improved SOCS3 proteomimetic scaffold with potential for targeting dysregulated JAK/STAT signaling in cancer.

## Graphical abstract

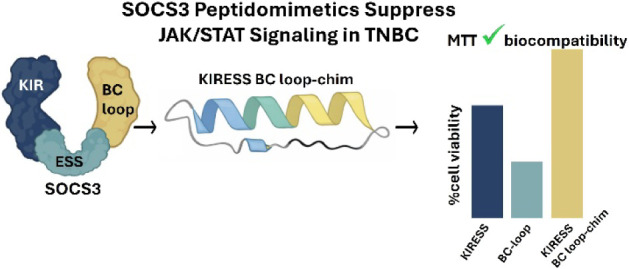

## Introduction

Aberrant expression of Suppressor of Cytokine Signaling 3 (SOCS3) protein has been associated with a broad range of pathological conditions, including autoimmune diseases, allergic responses, chronic inflammation, and cancer.^1^ By influencing cytokine receptor signaling, SOCS3 serves as a key regulator of immune balance and metabolic processes, positioning it as an attractive target for treating disorders rooted in immune and metabolic dysfunction.^2^ In the context of cancer, SOCS3 plays a context-dependent dual role, acting either as a tumor suppressor or promoter.^3^ It chiefly influences tumor development by modulating the Janus Kinase/Signal Transducer and Activator of Transcription (JAK/STAT) signaling pathway, which is frequently overactive in various types of cancer. In many cases, SOCS3 functions as a tumor suppressor by blocking key pathways involved in cell proliferation and survival.^4^ One mechanism involves its binding to STAT3, thereby preventing the activation of signals that drive cell growth. For instance, research has shown that restoring SOCS3 expression in lung cancer models can suppress tumor growth by downregulating Hypoxia-Inducible Factor 1-alpha (HIF-1α), a critical driver of angiogenesis and tumor expansion.^5^ In Non-Small Cell Lung Cancer (NSCLC), SOCS3 overexpression has been linked to smaller tumor size and increased apoptosis, reinforcing its tumor-inhibitory role.^6^ However, loss or silencing of SOCS3, often due to promoter hypermethylation, can enhance STAT3 phosphorylation, promoting unchecked cell proliferation and survival.^3,7,8^ This absence of regulation renders cells more responsive to growth signals mediated by the JAK/STAT pathway.^7^ SOCS3 acts as a critical tumor suppressor in breast cancer, including Triple-Negative Breast Cancer (TNBC), primarily by attenuating the Interleukin (IL)-6/JAK/STAT3 inflammatory signaling loop and related pathways that promote tumor growth, metastasis, and cancer stem cell maintenance. Therapeutic strategies aimed at restoring or mimicking SOCS3 function, or blocking IL-6/STAT3 signaling, show promise for managing aggressive TNBC subtypes.^9,10^

Given this, SOCS3-mimicking therapeutics, or proteomimetics, have emerged as promising tools for targeted therapy.^8^ Designing and creating molecules that imitate the binding or functional regions of proteins offers a powerful strategy for probing and regulating protein activity by selectively disrupting key molecular interactions. Among these, synthetic peptides have emerged as particularly effective mimetics, as they can replicate exact segments of proteins while also accommodating a wide array of chemical modifications. These include the use of non-canonical amino acids and alterations to the peptide backbone, enabling fine-tuned control over structure and function.^11^ In this study, we focus on chimeric constructs incorporating the KIR, ESS, and BC loop regions to evaluate cooperative effects on JAK2 binding. The design of peptidomimetics, including different regions of the same protein, is a rapidly advancing field: these compounds achieve improved specificity, affinity, and drug-like characteristics, offering promising tools for both basic research and therapeutic development.

The most common strategy for multiregion peptidomimetic design consists of the systematic analysis of the contribution of single peptides derived from various sequential and non-sequential regions of a protein to identify minimal binding epitopes, which are subsequently combined into a single peptidomimetic scaffold.^12^ Not many examples are available in the literature; thus, such designs could be highly innovative but would require substantial rational interface mapping and structural validation.^13^ Therefore, rational interface mapping and structural validation are essential to guide multi-epitope design.^14^

In the translation of potential anticancer compounds into drugs, to mitigate the side effects associated with non-targeted cancer therapies, a range of targeted drug delivery systems has been developed to address the specific biological and therapeutic requirements of the intended recipients. Of particular interest is the employment of several Cell-Penetrating Peptides (CPPs) for drug delivery, with a broad field of application such as gene therapy, nanotechnology, and diagnostics.^15,16^ CPPs have a variable number of residues^17^ often bearing positively charged amino acids (e.g., Arginine and Lysine) that interact with the negative charges of the membrane, leading to endocytosis as internalization mechanism^15^ as seen in the fragment 48–64 of the TAT (Transactivator of Transcription) protein of HIV-1.^18–22^ Several small CPPs, often identified through phage-display peptide library screening,^23^ have been reported, such as the sequence LTVSPWY.^24^

Our previous work led to the identification of KIRESS, a linear peptide encompassing SOCS3 protein residues 22–45, which includes both the Kinase Inhibitory Region (KIR) and the Extended SH2 Subdomain (ESS). This construct exhibited SOCS3-mimetic properties and demonstrated tumor-suppressive activity in squamous cell carcinoma models,^25^ as well as the ability to inhibit pulmonary metastases in TNBC.^20^ Subsequently, to enhance functional versatility, we engineered KIRCONG chim, a chimeric peptidomimetic combining non-contiguous SOCS3 regions linked via β-alanine spacers. This compound showed high binding affinity for the JAK2 catalytic domain and exerted pronounced anti-inflammatory and antioxidant properties in both Vascular Smooth Muscle Cells (VSMCs) and RAW 264.7 macrophages;^22^ however, it exhibited poor aqueous solubility. To address this, we developed an optimized analogue, KIRCONG chim PEG, in which the β-alanine linkers were replaced with a polyethylene glycol (PEG) moiety to enhance solubility. This peptidomimetic effectively inhibited STAT3 phosphorylation^26^ and, *in vivo*, significantly attenuated pathological retinal neovascularization in a murine model of Oxygen-Induced Retinopathy (OIR).^27^

Herein, by analyzing more in depth the crystallographic structure of the ternary complex between JAK2, Glycoprotein­(Gp)­130, and SOCS3 ^28^ we argued that another region, named the BC loop, after the CONG region, provides a series of hot spots in the complex formation. Thus, we designed several linear proteomimetics of SOCS3 (Figure 1), including or excluding the KIRESS region, and characterized them functionally and conformationally through MicroScale Thermophoresis (MST), Circular Dichroism (CD), and emission fluorescence investigations. In this study, we aimed to further improve affinity and stability by integrating the BC loop region into a chimeric construct (KIRESS BC loop-chim) and evaluating cooperative interactions with JAK2. Subsequently, they were analyzed in TNBC cell lines for their cellular compatibility.

**Figure 1:**
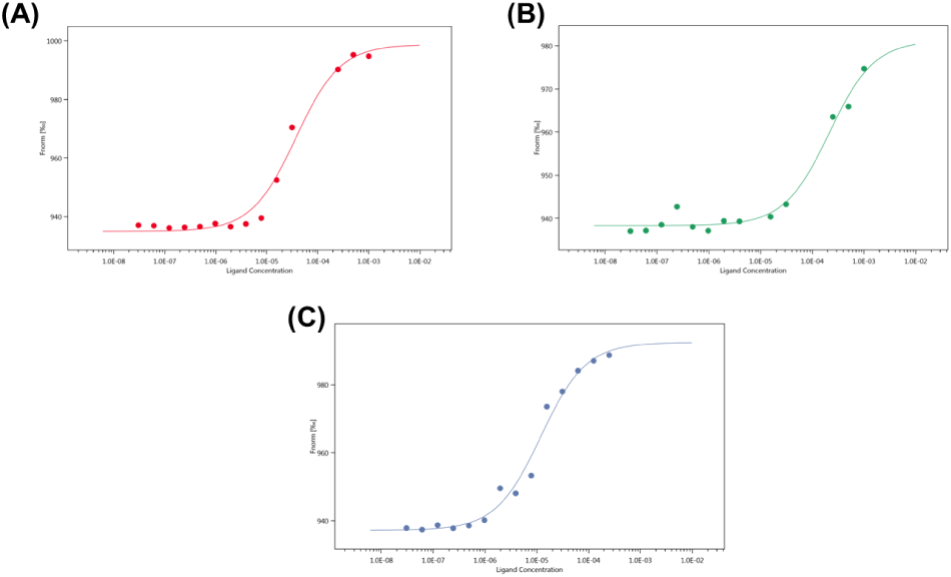
Binding isotherms demonstrate MST signals as a function of ligand concentration for (A) KIRESS, (B) BC loop, and (C) KIRESS BC loop-chim. Data represent the means of two independent experiments.

## Results and Discussion

### Design of Novel Peptidomimetics of SOCS3

A detailed analysis of the crystallographic structure of the ternary complex involving JAK2, Gp130, and the SOCS3 protein indicates that, in addition to the CONG, KIR, and ESS regions, the BC loop fragment also exhibits several key hot spots that contribute to the formation of the complex and could enhance cooperative binding.^28^ Indeed, the SOCS3 BC loop, which coordinates phosphotyrosine binding, also directly contacts JAK2. SOCS3 binds Gp130 via the canonical SH2 phosphotyrosine-binding groove, while the BC loop (Arg^71^–Phe^80^) engages in interactions with both Gp130 and JAK2 through opposing surfaces. Alanine-scanning mutagenesis identified Phe^25^ (KIR), Phe,^79^ and Phe^80^ (BC loop) as critical residues, with Asp^72^, Ser^73^, Phe^79^, and Phe^80^ of the BC loop directly contacting JAK2. On the basis of these considerations, several new linear peptides partially reproducing SOCS3 regions crucial for JAK2 interaction were designed (Table 1) separately or in conjunction. For the BC loop, in an attempt to reproduce the native structure, N-cap and C-cap motifs were added to stabilize the loop arrangement: N-terminal Ac-Trp-Ile^70^ and the C-terminal Gly-Thr-Trp-Ser^83^-Leu^82^-Thr^81^ peptides were added to form aromatic clusters.^29^ To study the involvement of the BC loop in JAK2 binding, we applied a previously established approach^22^ by designing a longer chimeric peptide that included the KIRESS and BC loop regions, named KIRESS BC loop-chim. In this design, to encourage loop turn formation, a Gly-d-Pro (Gp) motif was inserted as a conjunction between the two regions. In this chimeric peptide, the BC loop sequence is accommodated in a broader structured framework rather than forming an isolated loop identical to the BC loop peptide.

**Table 1:** Sequences of SOCS3 Peptidomimetics^a^

Name	Sequence	*K*_D_ (μM)
KIRESS	Ac-^22^LKTFSSKSEYQL^33^VVNAVRKLQESG^45^-NH_2_	(3.7 ± 0.6)*10
BC loop	Ac-WI^71^RDSSDQRHFF^80^TLSWTG-NH_2_	(2.1 ± 0.6)*10^2^
KIRESS BC loop-chim	Ac-^22^LKTFSSKSEYQL^33^VVNAVRKLQESG^45^ Gp ^80^FFHRQDSSDR^71^-NH_2_	11.0 ± 2.0

a Peptidomimetics analyzed in this study. Residues of the KIR region are reported in blue (22–33), ESS in orange (34–45), and the BC loop in green (71–80). Underlined residues were introduced to favor turn arrangements.

### Affinity toward the JAK2 Catalytic Domain of SOCS3 Mimetics

The binding affinity toward the catalytic domain of JAK2 of novel SOCS3 mimetics was evaluated through an MST assay.^30^ All compounds provided dose–response MST signals (Figure 1), whose fitting allowed the estimation of Dissociation Constant (K_D_) values (Table 1) of different magnitudes: in the case of KIRESS, *K*_D_ ∼ 40 μM; for the BC loop, *K*_D_ ∼ 200 μM; and for KIRESS BC loop-chim, *K*_D_ ∼ 11 μM.

For KIRESS, the affinity observed is comparable to that reported by us employing the Surface Plasmon Resonance (SPR) technique.^20^ The overestimation of the affinity in this latter case is likely due to covalent immobilization of the protein on the SPR-chip. Conversely, the peptide including the BC loop alone exhibited high micromolar *K*_D_. The modest affinity observed for the isolated BC loop peptide likely reflects the fact that this region contains only a few of the interaction hotspots contributing to SOCS3-mediated inhibition of JAK2. In the native protein, the KIR region provides the primary inhibitory interaction, while the BC loop contributes additional stabilizing contacts. Consequently, combining these elements in the chimeric construct allows cooperative engagement of multiple binding determinants.

Interestingly, the conjunction of these two regions in the KIRESS BC loop-chim mimetic increases the ability to bind to JAK2 with respect to separate regions, suggesting a cooperative effect between KIR/ESS and BC loop regions, likely via simultaneous engagement of multiple binding hotspots.

The *K*_D_ value obtained for the KIRESS BC loop-chim (∼11 μM) was among the lowest when compared with those previously reported by us for other SOCS3 peptidomimetics, including other regions besides KIR, as in the case of KIRCONG chim (*K*_D_ ∼ 12 μM)^22^ and was the most encouraging since the *K*_D_ value between SOCS3 and JAK2 is 0.1 μM.^29^

### Conformational Studies of SOCS3 Analogues

CD spectra of SOCS3 peptidomimetics were recorded in the far-UV region in 10 mM phosphate buffer and in the presence of two structuring agents, 2,2,2-trifluoroethanol (TFE) and sodium dodecyl sulfate (SDS), which are both used in studies to modulate the secondary structure of peptides.^31^ In Figure 2 (upper and medium panels), the overlay of CD spectra upon the increase of structuring agents is reported; their deconvolutions are presented in Tables S2 and S3.

**Figure 2:**
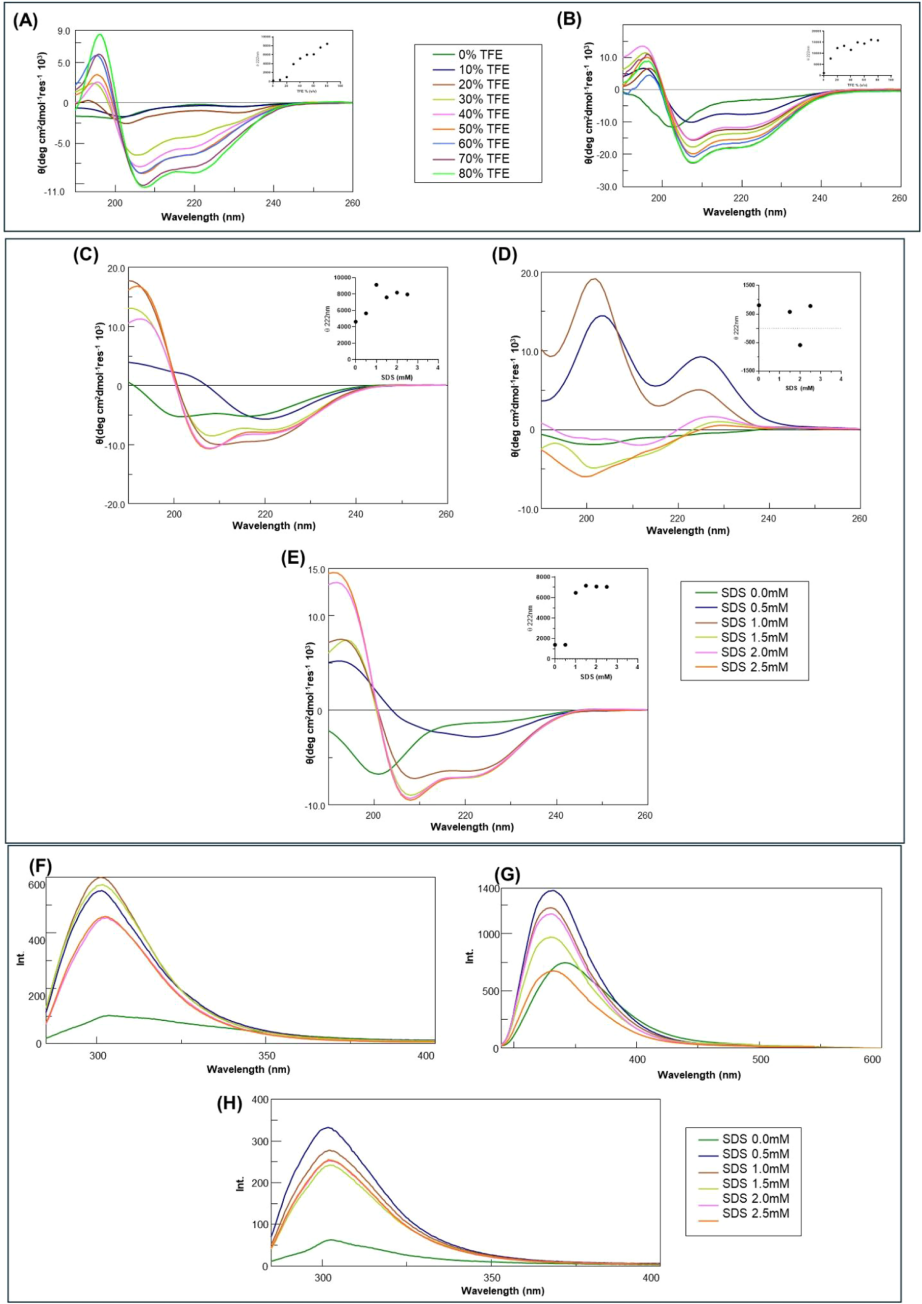
Conformational analysis of SOCS3 peptides. Overlay of CD spectra at increasing concentrations of TFE (upper panel) and SDS, (medium panel). Θ_222_ vs TFE percentages are reported as insets. Lower panel: overlay of fluorescence emission spectra at increasing concentrations of SDS. (A, D, G) BC loop; (B, E, H) KIRESS BC loop-chim; and (C, F) KIRESS.

In an aqueous buffer, short peptide fragments often lack sufficient intramolecular interactions to stabilize a defined secondary structure. Accordingly, the peptides analyzed here display CD spectra consistent with a predominantly random coil state in the absence of helix-inducing agents. The addition of TFE or SDS can stabilize intramolecular hydrogen bonding and promote the formation of α-helical structures.

For KIRESS, the TFE titration was already reported in ref. 20 where it exhibited a discrete tendency to assume a helical conformation, reaching its maximum content at ∼30% (v/v) of TFE. The BC loop (Figure 2A) and the chimeric KIRESS BC loop-chim (Figure 2B) also exhibited a similar propensity, which resulted in saturation only in the case of the chimeric peptide at ∼30% (v/v) TFE (Table S2). These results confirmed the ability of KIRESS to form its native conformation even when inserted into the chimeric construct.

In the case of SDS titration, only the chimeric KIRESS BC loop-chim (Figure 2E) exhibited a neat conformational transition from mixed (random + α) to helical conformations. Indeed, upon increasing SDS concentrations, the profile of the Θ_222_ value vs SDS concentration appeared monotonic, reaching a saturated value at 1.5 mM of SDS (Table S3). Conversely, KIRESS alone (Figure 2C) presented a less gradual structuring process: it exhibits an intermediate state (at 1.0 mM SDS) of coiled helices,^32,33^ which then disappeared until reaching a helical saturated conformation at 2.0 mM of SDS (Table S3).

The more pronounced helix-inducing effect observed for the KIRESS BC loop-chim peptide compared to KIRESS likely arises from the contribution of residues derived from the SOCS3 BC loop, which increase the overall amphipathic character and helix-forming propensity of the sequence. SDS provides a hydrophobic environment that mimics protein surfaces and can stabilize α-helical conformations. Under these conditions, the chimeric peptide undergoes a cooperative transition toward a more ordered helical structure, whereas KIRESS alone shows a weaker structural response due to the absence of the additional stabilizing interactions provided by the BC loop segment.

The SDS titration of the BC loop appeared quite different with respect to other mimetics, as it did not exhibit the stabilization of conformations. Indeed, at the two lowest SDS concentrations (0.5 and 1.0 mM), the presence of two positive bands centered at 204 and 202 nm, respectively, along with a band at 225 nm (Figure 2D) suggests the presence of loop arrangement due to the aromatic Trp zipper effect (Table S3).^34^ These are not standard β-sheet signals (which typically have a negative band at ∼215 nm) but are due to side-chain CD contributions. However, this effect is lost at higher SDS concentrations.

The CD analysis clearly indicated that TFE, which usually reduces water–peptide H-bonding and stabilizes intrapeptide H-bonds, induced a helical conformation in all three peptides, while in the presence of SDS, this tendency was confirmed only for KIRESS and KIRESS BC loop-chim. Indeed, SDS, which usually provides hydrophobic interfaces, in the case of the BC loop stabilizes a Trp-zipped hairpin/loop conformation. To deepen these conclusions, fluorescence spectra at increasing concentrations of SDS were registered (Figure 2, lower panel).

This analysis revealed distinct responses of KIRESS and KIRESS BC loop-chim in comparison to the BC loop, supporting differences observed in the CD results.

For KIRESS (Figure 2F), fluorescence intensity increased sharply from 0 to 1.0 mM SDS, then decreased at higher SDS levels, while the wavelength of the emission maximum (λ_max_) remained stable around 301–303 nm, indicating no significant spectral shift. In contrast, the BC loop (Figure 2G) exhibited a notable shift in λ_max_ (from 342 to 332 nm) between 0 and 0.5 mM SDS, then stabilized at ∼330 nm at higher SDS concentrations, suggesting a more hydrophobic environment, while the fluorescence intensity increased at 0.5 mM SDS with a decrease until 2.5 mM SDS, returning to baseline levels (Figure 2G). These changes are consistent with SDS-induced formation of a Trp-zipped β-hairpin conformation.^34^ The KIRESS BC loop-chim spectra showed a constant λ_max_ across SDS concentrations, but intensity reached its maximum value at 0.5 mM before gradually declining, suggesting concentration-dependent structural rearrangements without changes in the environment polarity (Figure 2H).^35^ Overall, these data confirm that SDS differentially affects the BC loop structure compared with the other peptides. Although TFE and SDS are artificial agents, combined CD and fluorescence data strongly support the formation of a loop in the chimeric construct, consistent with the intended structural motif.

### Cellular Assays: Stability and Biocompatibility

To evaluate the stability of SOCS3 mimetics in a cellular environment, a serum stability assay was conducted using 25% Fetal Bovine Serum (FBS). The reduction of peptide area percentages over time is reported in Figure 3 for the BC loop and KIRESS BC loop-chim. A similar experiment for KIRESS was already reported in ref 22.

**Figure 3:**
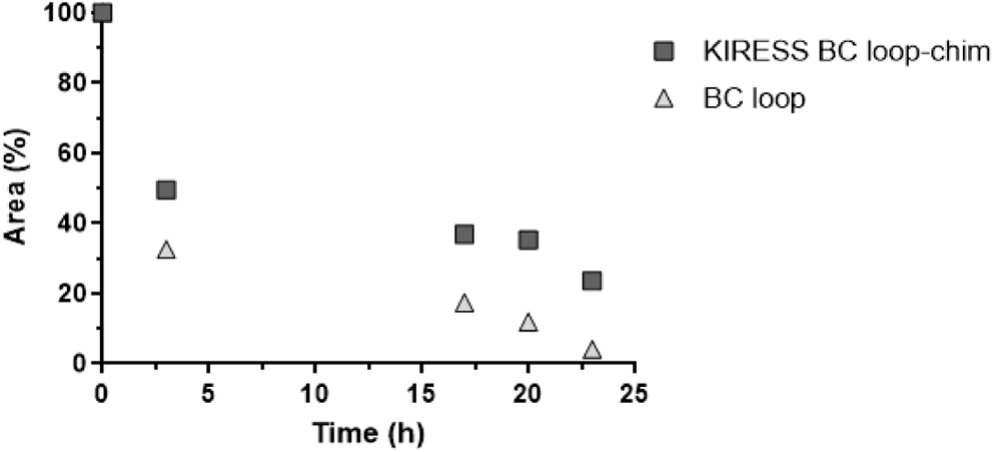
Serum stability of the BC loop and KIRESS BC loop-chim peptides. Peak area at *t* = 0 min was normalized to 100%; residual peak areas at later time points are reported as percentages relative to this baseline. Data are the mean of two independent experiments.

KIRESS BC loop-chim demonstrated significantly enhanced stability with respect to the BC loop over the course of 25 h. These findings suggest that the addition of the BC loop region into the broader structure of KIRESS BC loop-chim could compact the construct and thus reduce protease accessibility, potentially enabling prolonged intracellular activity. To allow cellular studies in MDA-MB-231 and MDA-MB-468 cells, the compounds were conjugated to the small CPP via a PEG_1_ linker (see Table S1). A negative control sequence (CTRL) was added.

TNBC cell lines were selected because the dysregulation of the JAK/STAT signaling pathway is frequently observed in aggressive breast cancers, making these models relevant for evaluating SOCS3-derived inhibitors targeting JAK2.^20,36^ The present experiments were intended as a preliminary assessment of peptide biocompatibility under disease-relevant conditions. Additional studies in non-tumorigenic cell lines (e.g., MCF-10A or HEK-293) will be valuable to further assess the safety profile of these constructs and will be considered in future investigations.

The cytotoxic effects of KIRESS, BC loop, KIRESS BC loop-chim, and CTRL peptides (Table S1) were evaluated after 24 and 48 h in both cell lines (Figure 4) at three different concentrations (12.5, 25, and 50 μM). The MTT assay showed that treatment with the tested compounds had minimal effects on the viability of both cell lines under the experimental conditions, with only slight reductions observed at specific concentrations, particularly for the BC loop peptide due to its limited water solubility. These results indicate that the compounds do not induce significant cytotoxicity, allowing further studies on their molecular interactions without confounding effects from cell death, which are currently ongoing.

**Figure 4:**
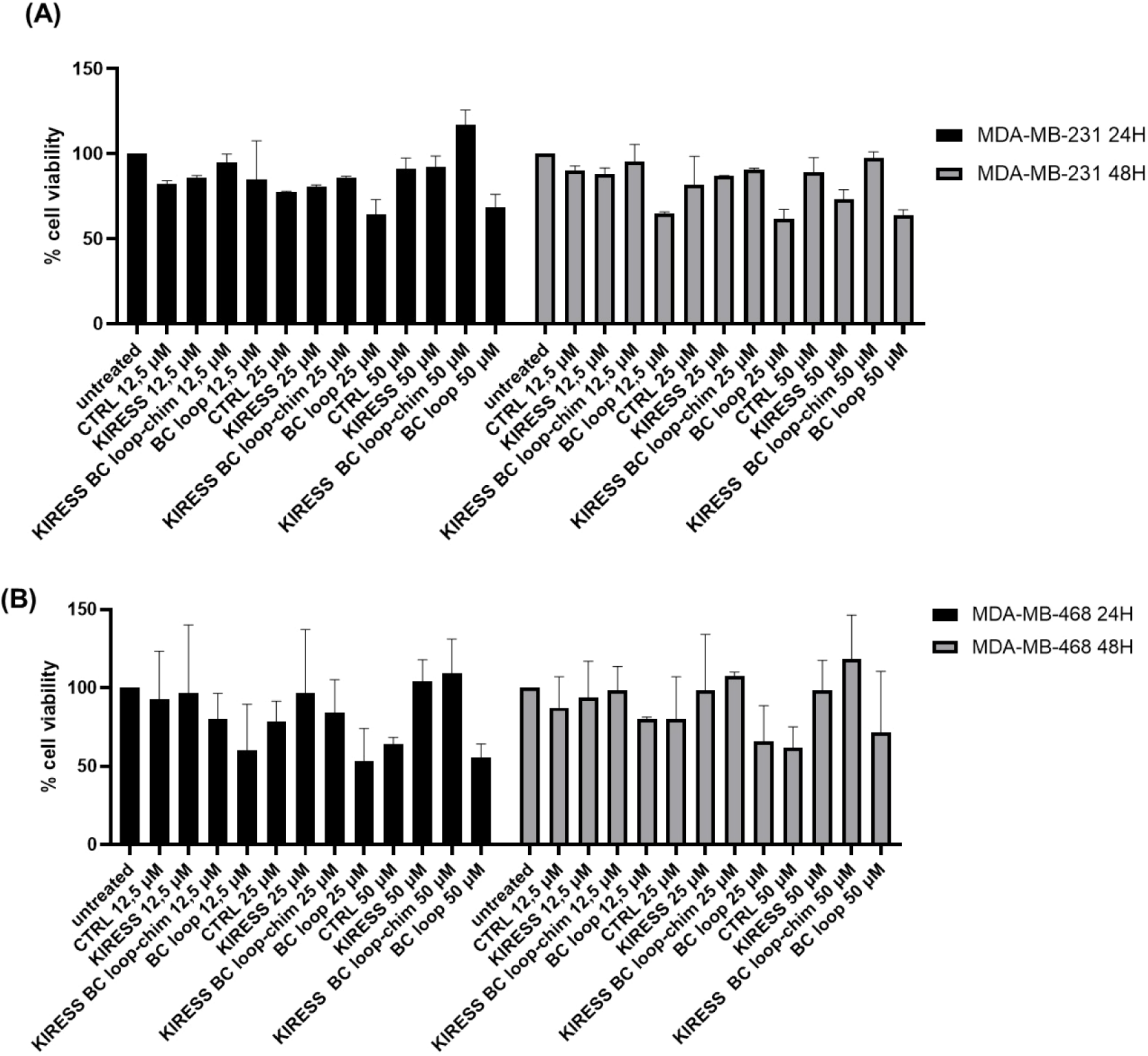
Effects of peptides on TNBC cell viability. MTT assay in (A) MDA-MB-231 and (B) MDA-MB-468 cell lines upon incubation with peptides at 24 and 48 h. Percentage of cell viability was assessed at increasing peptide concentrations. Statistical analysis was performed by the Kruskal–Wallis test followed by Dunn’s multiple comparisons test.

## Experimental Section

### Peptide Synthesis

The peptide synthesis reagents were obtained from Iris Biotech (Germany), while the solvents used for both peptide synthesis and High-Performance Liquid Chromatography (HPLC) analyses were sourced from Romil (Dublin, Ireland). The reversed-phase analytical columns and LC-MS system were supplied by Thermo Fisher Scientific (Waltham, MA, USA). Peptides were synthesized on a 50 μmol scale using the Fluorenylmethoxycarbonyl (Fmoc) strategy and standard Solid-Phase Peptide Synthesis (SPPS) protocols.^37^ Rink amide resin (loading: 0.67 mmol/g) was used as the solid support.^38^ Twenty-five μmol was used for *in vitro* characterization studies, while the remaining 25 μmol was conjugated to the CPP and a PEG_1_ spacer (Table S1). Of these, 10 μmol was further labeled with fluorescein for cellular uptake studies.^39^ The average crude yield of the peptides was approximately 80%. The final compounds were purified by Reverse-Phase HPLC (RP-HPLC), lyophilized, and stored at 4 °C until further use, yielding an average purification recovery of 70%.

### Microscale Thermophoresis

MST experiments were performed using a Monolith NT.115 system (NanoTemper Technologies), equipped with 60% LED and 40% infrared laser power. The His-tagged catalytic domain of JAK2 (residues 826–1132; Carna Biosciences) was labeled with the His-Tag Labeling Kit RED-tris-NTA (NanoTemper Technologies), as previously described.^40^ The final concentrations were adjusted to 100 nM for the dye and 200 nM for the protein. Equal volumes (100 μL) of protein and dye solutions were mixed and incubated in the dark for 30 min at room temperature. KIRESS, BC loop, and KIRESS BC loop-chim peptides were prepared from 2 mM stock solutions in a labeling buffer (NanoTemper Technologies). To evaluate the binding of these SOCS3 analogues to JAK2, 1:1 serial dilutions were carried out, typically yielding 14–16 samples.^22^ Measurements were performed at 25 °C in standard capillaries (NanoTemper Technologies) using an assay buffer composed of 50 mM Tris-HCl, 150 mM NaCl, 0.05% Triton X-100, 1 mM dithiothreitol (DTT), and 10% glycerol, pH 7.5. Binding affinities were determined using the MO Affinity Analysis software (MO-S002; NanoTemper Technologies), employing the fitting model provided by the manufacturer. The binding data at varying concentrations were fitted using the equation implemented in the MO Affinity Analysis software,^41^ as reported below:

$$f\left(c\right)=\mathrm{Unbound}+\frac{\left(\mathrm{Bound}-\mathrm{Unbound}\right)\times c+c\left(\mathrm{target}\right)+K_{D}-\sqrt{{\left(c+c\left(target\right)+K_{D}\right)}^{2}-4c\times c\left(target\right)}}{2c\left(target\right)}$$

The equation is derived from the Langmuir binding isotherm. In this model, “*f*(*c*)” represents the fraction of bound complex at a given peptide concentration “(*c*)”. The parameter “Unbound” refers to the F_norm_ (normalized fluorescence) signal or the raw fluorescence signal in the initial fluorescence mode of the free target protein (JAK2). “Bound” corresponds to the signal of the JAK2–peptide complex. The final concentration of the target in the assay is denoted as “*c*(*target*)”.

### Circular Dichroism (CD) Spectroscopy

CD spectra were recorded using a Jasco J-815 spectropolarimeter (JASCO Corp., Milan, Italy) at 25 °C in the far-UV region (190–260 nm). The BC loop and KIRESS were analyzed at a 100 μM concentration^20^ while KIRESS BC loop-chim was analyzed at 50 μM in a 10 mM phosphate buffer at pH 7.4, with and without TFE (from 0 to 80%) or SDS (from 0.0 to 2.5 mM), by employing a quartz cuvette (0.1 cm path length). Blank spectra were subtracted from each recorded spectrum. The deconvolution of CD spectra was performed using the BESTSEL software (http://bestsel.elte.hu/).

### Fluorescence Assays

Fluorescence assays were performed on a Jasco FP-8300 spectrofluorometer with a 1.0 cm path-length quartz cuvette. The peptide concentrations were 50 μM for BC loop and KIRESS, and 25 μM for KIRESS BC loop-chim. Fluorescence spectra were recorded in the absence or presence of different concentrations of SDS (0.0, 0.5, 1.0, 1.5, 2.0, 2.5 mM) in 10 mM phosphate buffer (pH 7.4) at 25 °C under magnetic stirring. For BC loop λ_exc_: 280 nm, λ_em_: 290–400 nm; for KIRESS and KIRESS BC loop-chim λ_exc_: 275 nm, λ_em_: 285–400 nm.

### Serum Stability

Peptides were mixed with 25% (w/v) Fetal Bovine Serum (FBS) to a final concentration of 1 mg/mL and incubated at 37 °C.^26^ Aliquots of 50 μL were collected at various time points: 0, 3, 17, 20, 23, and 42 h. Each sample was treated with 50 μL of 30% trichloroacetic acid (TCA) and incubated at 2 °C for at least 15 min to precipitate serum proteins. Following centrifugation at 13,000 rpm for 15 min, the supernatant was collected. Samples were analyzed using RP-HPLC on a Jasco LC-4000 series HPLC system equipped with a UV detector and a Phenomenex C18-Kinetex column (Milan, Italy). Gradient elution was carried out at 25 °C, monitored at 210 nm, using buffer A (0.1% trifluoroacetic acid (TFA) in H_2_O) and buffer B (0.1% TFA in acetonitrile), with a linear gradient from 5% to 70% buffer B over 20 min. The areas under the peaks corresponding to the recovered compounds were integrated over time, using the peak area at *t* = 0 as the 100% reference.

### Cells

MDA-MB-231 and MDA-MB-468 cells were cultured in Dulbecco’s Modified Eagle Medium (DMEM) (GIBCO, Paisley, UK) supplemented with 2 mM l-glutamine, 50 ng/mL streptomycin, 50 units/mL penicillin, and 10% heat-inactivated Fetal Bovine Serum (FBS) (GIBCO). Cells were maintained in a humidified atmosphere with 5% CO_2_ at 37 °C, and once they reached 70–80% confluence were harvested with 0.25% trypsin (Sigma-Aldrich, St Louis, MO, USA) as previously described^42^ and used for the experiments.

### 3-[4,5-Dimethylthiazol-2-yl]-2,5-diphenyl tetrazolium bromide (MTT) Viability Assays

MTT viability assay was performed on cell lines following treatment with peptides. MDA-MB-231 and MDA-MB-468 cells (5 × 10^3^ cells/well) were seeded in duplicates in 96-well plates and allowed to adhere for 24 h. The peptides were initially dissolved in DMEM; however, the BC loop demonstrated limited solubility in this medium, a property likely attributable to the presence of multiple aromatic residues. Consequently, stock solutions (2 mM for KIRESS and KIRESS BC loop-chim, and 500 μM for BC loop) were prepared in water and then diluted in the culture medium to reach the desired final concentrations. To ensure that the presence of the solvent did not influence the assay outcome, an equivalent volume of sterile water was added to the control wells lacking peptide. Peptides were added to the cell cultures at increasing concentrations (12.5, 25, and 50 μM). After 24 h and 48 h of incubation, 10 μL per well of MTT solution (Sigma-Aldrich) were added for 4 h. Then, 150 μL of stop solution of dimethyl sulfoxide (DMSO) were added to each well, and absorbance was read at 570 nm using the Glomax Discover Microplate Reader (Promega, Madison, WI, USA).

## Conclusions

The present work expands the design principles of peptidomimetics of SOCS3 by integrating a third structural determinant of the SOCS3/JAK2/Gp130 interface, the BC loop, into new constructs that complement the well-established KIR and ESS regions. Structural inspection of the crystallographic complex indicates that the BC loop contributes additional hot-spot interactions, prompting the synthesis of individual and chimeric mimetics to assess its functional relevance.

The adopted strategy is based on a structure-guided analysis of the SOCS3/JAK2/Gp130 interface and incorporates residues from the BC loop, a complementary interaction region. This design enables cooperative engagement of multiple binding determinants and contributes to the improved affinity observed for the chimeric construct. Biophysical analyses established that the isolated BC loop peptide binds JAK2 with a modest affinity (*K*_D_ ∼ 200 μM), significantly weaker than the KIRESS peptide. However, incorporating the BC loop region into KIRESS BC loop-chim produced a marked improvement in affinity (*K*_D_ ∼ 11 μM). These findings underscore that full recapitulation of the SOCS3 interface requires simultaneous presentation of multiple noncontiguous motifs and that cooperative effects between these regions can be reestablished through rational peptide engineering. The cooperative effect observed in the KIRESS BC loop-chim likely arises from the simultaneous interaction of KIR/ESS and BC loop regions with multiple hot spots on JAK2, stabilizing the interface and enhancing binding affinity. Compared to our previous KIRCONG constructs, KIRESS BC loop-chim offers advantages in affinity (∼11 μM vs 20–40 μM) and serum stability. Although the affinity of the chimeric peptide (∼11 μM) remains approximately 2 orders of magnitude weaker than that of the native SOCS3 protein (∼0.1 μM), this difference is expected because the isolated peptide lacks the extensive interaction surface and structural stabilization provided by the full protein scaffold.

Conformational analyses support these considerations: the isolated BC loop displayed a Trp-zip-induced loop arrangement only at low SDS concentrations, whereas the chimeric peptide KIRESS BC loop maintained a defined secondary structure under both TFE- and SDS-induced conditions. The pG turn and the aromatic capping motifs likely contribute to stabilizing a native-like loop arrangement, enabling the chimeric peptide to present its hot-spot residues in a conformation more compatible with the JAK2 interface. In contrast, KIRESS alone retained its previously reported helical propensity, confirming that both regions preserve their folding behavior when embedded in the chimeric scaffold. Future studies could include solution NMR or hydrogen–deuterium exchange mass spectrometry to confirm conformation stability. The enhanced structural stability of the KIRESS BC loop-chim correlated with improved serum stability relative to the BC loop peptide. Given that metabolic liability is a persistent challenge for peptide-based inhibitors of intracellular pathways, the observed stabilization is a notable advantage for translational progression. Future studies should address the selectivity among JAK family kinases.

## Supplementary Material



## Notes

Sequences of SOCS3 peptidomimetics assayed in cells (Table S1). Deconvolution of CD spectra upon TFE titration (Table S2). Deconvolution of CD spectra upon SDS titration. (Table S3) (PDF)

## Abbreviations

CD: Circular Dichroism
DMEM: Dulbecco’s Modified Eagle Medium
DTT: Dithiothreitol
ESS: Extended SH2 Subdomain
FBS: Fetal Bovine Serum
JAK: Janus Kinase
KIR: Kinase Inhibitory Region
MTT: 3-[4,5-Dimethylthiazol-2-yl]-2,5-diphenyl tetrazolium bromide
MST: MicroScale Thermophoresis
PBS: Phosphate-Buffered Saline
SDS: Sodium Dodecyl Sulfate
SOCS: Suppressor of Cytokine Signaling
SPPS: Solid Phase Peptide Synthesis
STAT: Signal Transducer and Activator of Transcription
RP-HPLC: Reverse Phase-High Performance Liquid Chromatography
TFA: Trifluoroacetic Acid
TFE: 2,2,2-Trifluoroethanol
TNBC: Triple-Negative Breast Cancer
