# Supplementary material for: Structure-Guided SOCS3 Peptidomimetics: Design and Functional Characterization
Source: ACS Omega. 2026 Apr 13;11(16):24739–47. doi: 10.1021/acsomega.6c01336 (PMC13129812; doi:10.1021/acsomega.6c01336)
Supplement: Supplementary file 1 [file ao6c01336_si_001.pdf]

Structure-guided SOCS3 Peptidomimetics: design and functional characterization

*Alessia Cugudda [a], Sara La Manna [a], Candida Bucciero [b], Giuliano Castellano [b], Anna Maria Malfitano [b], and Daniela Marasco [a, *]*

[a]Department of Pharmacy - University of Naples Federico II, 80131, Naples, Italy

[b]Department of Translational Medical Sciences, University of Naples Federico II, 80131, Naples, Italy.

*Corresponding Author Email: daniela.marasco@unina.it Phone: (+39)081-2534512

Table S1. Sequences of SOCS3 peptidomimetics assayed in cells, Polyethylene glycol (PEG_1) and Cell Penetrating Peptide (CPP).

**Name**	**Sequence**
KIRESS	Ac-LTVSPWY-PEG_1-^{22}**LKTFSSKSEYQL**^{33}**VVNAVRKLQESG**^{45}
BC-loop	Ac-LTVSPWY-PEG_1-W^{70}**IRDSSDQRHFFTL**^{82}SWTG
KIRESS BC loop-chim	Ac-LTVSPWY-PEG_1-^{22}**LKTFSSKSEYQL**^{33}**VVNAVRKLQESG**^{45} G p ^{80}**FFHRQDSSDR**^{71}
CTRL	Ac-LTVSPWY-PEG_1-$(RAA)_{10}$

Peptide sequences with CPP+PEG_1 added (underlined). Residues of KIR region are in blue (22–33), ESS orange (34–45) and BC-loop in green (70–82).

Table S2. Deconvolution of CD spectra upon TFE titration.

TFE	α-helix	β-sheet	Turn	Others
0	0	37.7	14.6	47.6
10%	0	37.5	14.4	48.1
20%	0	34.3	14.9	50.8
30%	11.8	22.9	14.6	50.7
40%	15.8	20.7	12.9	50.6
50%	18.9	17.4	13.2	50.5
60%	20.1	18.1	12.3	49.5
70%	22.5	13.1	11.8	52.2
80%	28.3	10.5	10.3	50.9

Deconvolution of CD spectra of BC loop.

% TFE	α-helix	β-sheet	Turn	Others
0	0.4	26.5	16.6	56.4
10	23.3	13.8	14.3	48.7
20	30.8	13.8	10.8	44.6
30	34.2	10.5	10.0	45.3
40%	34.5	7.7	11.5	46.4
50%	34.7	12.0	8.4	44.9
60%	30.7	14.9	9.3	45.2
70%	35.9	12.3	8.4	43.4

80%	35.1	12.9	8.4	43.6

Deconvolution of CD spectra of KIRESS BC loop-chim.

Table S3. Deconvolution of CD spectra upon SDS titration.

SDS (mM)	α-helix	β-sheet	Turn	Others
0 .0	3.7	23.1	20.2	53.0
0.5	1.2	39.5	13.9	45.5
1.0	30.4	18.8	15.2	35.5
1.5	37.3	8.4	12.0	42.3
2.0	43.7	4.5	12.8	39.0
2.5	44.0	3.7	12.7	39.6

Deconvolution of CD spectra of KIRESS BC loop-chim.

SDS (mM)	α-helix	β-sheet	Turn	Others
0 .0	5.4	25.4	14.7	54.4
0.5	0.0	36.9	15.0	48.1
1.0	20.7	28.2	11.3	39.7
1.5	48.6	5.1	9.9	36.5
2.0	41.7	5.6	9.9	42.9
2.5	45.0	0.6	9.6	45.0

Deconvolution of CD spectra of KIRESS.

SDS (mM)	α-helix	β-sheet	Turn	Others
0 .0	1.5	37.8	16.1	44.6
0.5	0.0	79.3	15.8	4.9
1.0	0.0	84.9	10.4	4.6
1.5	4.9	33.5	11.0	50.6
2.0	1.3	43.0	11.0	44.7
2.5	3.9	30.9	15.1	50.1

Deconvolution of CD spectra of BC loop.
